# Comparing the Relationship Between Social Determinants of Health and Frailty Status of Medicare Beneficiaries in Rural and Urban Areas in the United States

**DOI:** 10.3390/jal6010006

**Published:** 2026-01-09

**Authors:** Hillary B. Spangler, David H. Lynch, Wenyi Xie, Nina Daneshvar, Haiyi Chen, Feng-Chang Lin, Elizabeth Vásquez, John A. Batsis

**Affiliations:** 1 Division of Geriatric Medicine, UNC School of Medicine, Chapel Hill, NC 27599, USA; 2 Department of Biostatistics, Gillings School of Global Public Health, University of North Carolina at Chapel Hill, Chapel Hill, NC 27599, USA; 3 Department of Epidemiology and Biostatistics, College of Integrated Health Science, University at Albany, Albany, NY 12222, USA; 4 Carolina Population Center, University of North Carolina at Chapel Hill, Chapel Hill, NC 27516, USA; 5 Department of Nutrition, Gillings School of Global Public Health, University of North Carolina at Chapel Hill, Chapel Hill, NC 27599, USA

**Keywords:** frailty, social determinants of health, rural, unmet needs, older adults

## Abstract

**Background:**

Frailty is a geriatric syndrome of increased physiological vulnerability, decreasing an older adult’s ability to successfully cope with health stressors. Social determinants of health (SDOH), including rural residence, can amplify healthcare disparities for older adults due to less accessibility to resources and lead to worse health outcomes. While the impact of rurality on older adult health is well-established, little is known about how the interaction of SDOH and geographical residence impact frailty status in older adults.

**Methods:**

Older adults (65+ years) in the National Health and Aging Trend Study (2011–2021) were categorized using Fried’s frailty phenotype (robust, pre-frail, frail). Rurality was defined using the 2013 Rural–Urban Continuum Codes. Generalized estimation equations with generalized logit link function determined the relationship between SDOH (healthcare access, community support, income, education) and frailty status.

**Results:**

Of *n* = 6082 participants (56.4% female), the mean age was 75.12 years (SE 0.10), 1133 (18.6%) lived in rural residence, and 2652 (53.0%) had pre-frailty. Although there was no relationship between geographical residence and frailty status (*p* = 0.73), we did observe lower associated odds of worse frailty status for those with Medigap insurance coverage (0.81, SE 0.08; *p* = 0.04) and inconsistent frailty status trends for those of divorced (1.12, SE 0.05; *p* = 0.007) and never married (0.20, SE 0.03; *p* < 0.001) status in urban areas.

**Conclusions:**

Our findings suggest that geographic residence may modify the relationship between SDOH and frailty status in older adults, providing novel insight into the complexity of these interactions. This work is important for identifying modifiable areas where additional support interventions may be important for mitigating frailty development and progression for older adults with efforts at both the individual and system levels.

## 1. Introduction

Frailty is a geriatric syndrome of increased physical vulnerability, decreasing an older adult’s ability to successfully cope with health stressors [1,2]. Frailty increases an older adult’s risk of morbidity and mortality and increases their risk of hospitalization and long-term nursing home placement [3,4]. Older adults living in rural areas may experience more pronounced effects of frailty such as functional decline, long-term nursing home placement, and increased mortality due to unique challenges associated with social determinants of health (SDOH) linked to rural living [5-7]. While geographical residence can be considered a SDOH, less is known about how the interaction between SDOH (education level, income level, healthcare access, community support) and rurality can impact the frailty status and downstream health status of community-dwelling older adults [8-11].

Understanding the complex relationship between SDOH and rurality has important implications for informing both individual- and policy-level health resource allocation for older adults with unmet needs. While clinical factors may be more easily acknowledged as contributors to frailty, there is still a gap in our understanding of how living in different geographical areas can impact frailty development. Specifically for rural areas, ecological factors such as increased travel distances to healthcare, limited home health agency access, rural hospital closures, and disjointed support systems may further exacerbate or quicken frailty development [12]. Frailty status is thought to be dynamic, with the potential for improvement or progression, with a pre-frailty state having a higher likelihood of reversibility than those with frailty [1,13]. Due to the potential for reversibility of prefrailty in community-dwelling older adults, it is important to understand the ecological characteristics that can impact frailty status [13,14].

Understanding the clinical and ecological contributing factors to frailty lends to the goal of precision medicine: the right treatment based on the individual’s characteristics [15]. This approach acknowledges that addressing the heterogeneity of aging requires tailored solutions rather than a “one size fits all” model. Therefore, we aim to assess the prevalence of frailty phenotypes in rural versus urban areas and identify how rurality modifies the relationship between SDOH and frailty status in community-dwelling older adults in the United States. We hypothesize a higher prevalence of worse frailty states in rural areas, with rurality strengthening the impact of SDOH on frailty status. This work will provide guidance to address the unmet needs of older adults with frailty with future interventions at both individual and system levels.

## 2. Materials and Methods

### 2.1. Study Participants

We used a nationally representative dataset, the National Health and Aging Trends Survey (NHATS), that contains a longitudinal cohort of Medicare beneficiaries (*n* = 8245). NHATS includes participants aged 65 years and up, and we focused on the data collection years from 2011 (Round 1) to 2021 (Round 11). Participants were selected for recruitment in 2011 via scientific sampling procedures as outlined in NHATS recruitment protocol [16]. Participants were included if they were a community-dwelling older adult and had complete data for frailty classification as discussed in 2.2 (*n* = 6082). If the participant needed assistance with or was unable to perform the interview, a proxy completed the interview. Participants were excluded if they resided in a nursing home or residential care, as these individuals are thought to receive more support at baseline. We received institutional review board approval from the Institutional Review Board of the University of North Carolina-Chapel Hill (23-1085) to conduct the secondary analysis. The study design, variable choice, and study sample are also reflected in our previous work examining frailty trajectories, which can be reviewed here: https://www.mdpi.com/2673-9259/5/3/27 (accessed on 15 October 2025) [17].

### 2.2. Frailty Phenotypes

Using Fried’s frailty phenotype (unintentional weight loss, low physical activity, slowness, weakness, exhaustion), we categorized frailty phenotypes based on the scoring system: robust = no items, pre-frail = 1 or 2 items, and frail ≥ 3 items [1]. Fried’s frailty phenotype is a common method of clinical frailty assessment [18,19]. All components of Fried’s frailty phenotype were available in each round of the study. If the participant had 4 of 5 missing frailty criteria, they were removed from the study.

### 2.3. Social Determinants of Health

#### 2.3.1. Residential Status

Residential status was defined based on the 2013 Rural–Urban Continuum Codes (RUCCs). RUCCs include two categories of residential designation: metropolitan (three codes) and non-metropolitan (six codes) indicators at the time of the intake interview [20]. Based on the previous literature using NHATS publications using RUCCs, we similarly considered metropolitan as urban and non-metropolitan as rural interchangeably in our analysis and discussion [21,22].

#### 2.3.2. Healthcare Access

Healthcare access was defined by multiple binary (yes/no) variables, including seeing a doctor within the last year (in-person or virtual), having a primary doctor, and receiving transportation assistance to the medical visit. We also included the participant’s insurance payer in addition to Medicare (e.g., Medigap and Medicaid).

#### 2.3.3. Income

Income was self-reported by the participant, ranging from $0–200,000 and above. These were broken into categories in order to provide more specific characteristics of the group.

#### 2.3.4. Education

Education level was also self-reported by the participant. Possible categories included less than high school, high school, some college, or college degree or higher.

#### 2.3.5. Support System/Community

Support system/community was defined by three variables measuring the amount of support for the participant (total hours of help per month), support in accessing healthcare (help with transportation to the doctor), and possible household support (household size). We also considered marital status to be a marker of support system/community.

### 2.4. Covariates

Covariates included self-reported race, ethnicity, sex, primary language, smoking and alcohol use, employment status, and multiple chronic conditions. Multimorbidity was defined as self-reporting two or chronic diseases out of a list of nine diseases including cardiovascular disease, high blood pressure, arthritis, osteoporosis, diabetes, lung disease, stroke, cancer, and dementia [23].

### 2.5. Statistical Methods

Baseline demographics and frailty status were analyzed using descriptive statistics, including means and standard errors (SEs) for continuous variables and frequencies and percentages for categorical variables. The comparisons between urban and rural areas employed *t*-tests for continuous variables and Chi-squared tests for categorical variables in the 2011 cohort. To examine changes in frailty status over time, we used generalized estimating equations (GEEs) with a multinomial logistic link to account for repeated frailty measurements taken from the same individuals across multiple study rounds. GEEs also accounted for participants who died or were no longer community-dwelling at any point in the study [24]. Time was modeled as years since baseline interview, allowing us to capture how frailty trajectories evolved over the follow-up period. All demographic characteristics and SDOH variables were measured at baseline and treated as unchanged in the models. The models included interactions between the rural/urban status and both baseline SDOH variables and time, which allowed us to assess whether the associations between SDOH and frailty differed by geographic location and whether rural–urban differences in frailty changed over the follow-up period. As noted above, we controlled for the covariates previously listed to minimize confounding between the covariates and frailty status. Missing data were addressed by using the missForest algorithm, except for income and frequency of help, due to a high degree of missingness. NHATS sampling weights were used to simulate the sample representation of the United States population.

All analyses were performed using SAS 9.4 (Cary, NC, USA) and R 4.3.3 (Vienna, Austria).

## 3. Results

Table 1a,b present the demographic and baseline characteristics of the 2011 cohort stratified by residential location (rural versus urban). Table 1a,b are also representative of the sample used in our previous frailty trajectory work [17]. Table A1 shows the association between baseline SDOH and frailty status without rural interaction term (*n* = 6082). There were *n* = 6082 participants from 2011 to 2021, with a mean age of 75.12 (SE = 0.10) and 56.4% and 81.2% of the sample female and white, respectively (Table 1a). Of the 6082 participants, 1133 (18.6%) were in a rural residence and there were no significant relationships between multimorbidity and geographical residence (*p* = 0.98) (Table 1b). Frailty statuses did not significantly differ between rural and urban areas (*p* = 0.73), with most participants having pre-frailty (53.0%). Comparatively, older adults had a higher prevalence of frailty in rural areas (25.3% versus 23.7%) and had a higher prevalence of pre-frailty in urban areas (53.3% versus 51.9%) (Table 1b). Generally, older adults in urban areas had higher education and income levels, reaching statistical significance (*p* < 0.05) (Table 1a). Older adults in rural areas had higher marriage rates than in urban areas (58.2% versus 53.8%, *p* = 0.01).

### 3.1. The Relationship Between Baseline SDOH, Geographical Residence, and Frailty Status

Table 2 shows how geographical status modifies the relationship between baseline SDOH (year 2011) and frailty status from 2011 to 2021. In urban areas, there were generally lower odds of frailty (versus pre-frailty and robust) for most age categories, with higher odds of pre-frailty (versus robust) in older adults ages 70–79 years old. Additionally, female sex was associated with lower odds of pre-frailty and frailty in urban areas.

### 3.2. Healthcare Access

Receiving help with transportation to a doctor and having a regular doctor were associated with lower odds of pre-frailty and frailty (Table 2). On average, having Medigap insurance in urban areas was associated with lower odds of frailty and pre-frailty, all reaching statistical significance. However, an opposite and statistically significant trend was found in older adults in urban populations with Tricare (available to those with military service affiliations), who had increased odds of pre-frailty, yet a decreased odds of frailty.

### 3.3. Support System/Community

Separated/divorced status was associated with higher odds of pre-frailty than married status (Table 2). Never married and living with a partner statuses were associated with lower odds of pre-frailty and frailty for older adults in urban areas; however, these groups had small sample sizes (Table 1a).

### 3.4. Education and Income

Living in an urban area with higher than a college-level education was associated with a higher odds ratio of frailty and pre-frailty than rural residents (Table 2). Lower income levels in urban areas ($25,000–74,999) were associated with higher odds of frailty.

## 4. Discussion

Our study suggests geographical residence modifies the relationship between baseline SDOH and frailty status. Even though there was no relationship between geographical residence and frailty status, we did observe modified relationships between specific baseline SDOH and frailty status when accounting for geographic residence. Urbanicity was associated with lower odds of worse frailty states for older adults with community/companion support and Medigap insurance coverage. These findings provide novel insight into the complex interaction between SDOH and geographical residence and their potentially lasting influence on older adult frailty status. Our findings are the first step in identifying gaps in the current healthcare infrastructure, where additional support interventions, at both the individual and policy level, may have a high impact on mitigating frailty development and progression.

Surprisingly, contrary to our hypothesis that rurality would be associated with worse multimorbidity and frailty statuses, there was no significant difference in frailty status between rural and urban residence (Table 1). This may be explained by the similar percentages of older adults with pre-frailty in rural and urban areas in this dataset and may represent a bias through the overrepresentation of older adults with better health status able to participate in a research study and/or differences in health behaviors not captured in this analysis [25]. Unexpectedly, we observed lower odds of worse frailty statuses for female sex. There is substantial literature showing that females typically experience more severe frailty states than males, yet have longer survival periods [14,26]. We suspect this is related to the geographic definitions for this particular sample, which may be overshadowing a confounder not currently being controlled for.

Support system/community support, such as being married and receiving assistance to medical appointments, was associated with lower odds of pre-frailty and frailty. Our results are consistent with the previous literature surrounding the impact of “social frailty” on health outcomes, where older adults with social/supportive networks experience less frailty and those of divorced and widowed status can be associated with worse health, cognitive impairment, and potentially frailty [27-31]. This concept was further supported by our findings of increased odds of pre-frailty and frailty associated with separated/divorced and widowed status with and without the urban geographical modifier (Tables 1 and A1). We unexpectedly observed worse frailty status for those living with their partner, which may be because of smaller sample sizes or reverse causality theory, where for older adults, living with spouses with poor health may negatively impact their own health [31,32]. Notably, urbanicity was associated with lower odds of pre-frailty and frailty for older adults that never married or lived with a partner, which may suggest a difference in access to external support between rural and urban areas, allowing older adults to remain in their homes as they age [33]. Our results suggest the importance of social support/community as a potential method for frailty mitigation, focusing efforts on areas with less societal or healthcare infrastructure for support resources.

Based on our results, healthcare infrastructure support can be impacted by insurance coverage, which can impact the degree of support for older adults based on their healthcare plan enrollment. We observed older adults with Medigap in urban areas having lower odds of frailty in all groups, reaching statistical significance. Because Medigap tends to enroll those of higher socioeconomic status, these findings align with the previous literature noting generally better health outcomes of older adults with Medigap insurance and residing in less rural areas [34].

A strength of our study lies in examining how geographical residence can modify the relationship between baseline SDOH and older adult frailty status, which has not been extensively explored in the literature. Our study is clinically relevant, as SDOH, including geographical residence, can impact clinical care approaches aimed at slowing the progression of frailty. The limitations of our study include its secondary analysis design, limiting the causality of the observed relationships, and its susceptibility to the impact of missing data, which we attempted to best address by using multiple imputation methods. We also were limited by small sample sizes within certain categories, possibly leading to type II error and large changes in effect size and direction. Yet, it was felt important to have representation for age brackets of 85 years and up and Hispanic ethnicity, as these groups are typically under-represented in research. We also want to acknowledge that there are societal structural biases that may impact our results that we are not fully able to account for, and our SDOH definitions may not adequately capture lived experiences While Fried’s frailty phenotype is a good clinical measure of frailty, there is a risk of incurring ceiling and floor effects of the scale, which may impact accurately capturing the frailty status of individuals on extremes of scoring (e.g., 0 or 5 frailty items). Similarly, our effects are at risk of survival bias, as those dying prior to the end of the study may be significantly different than the studied sample. NHATS attempts to provide a representative sample of older adults in the United States; however, our results may not be applicable to older adults that are less likely to participate in research studies due to SDOH. Notably, we did not assess collinearity between sociodemographic and SDOH covariates because all are thought to be clinically impactful; however, this may present unintentional confounding. Lastly, because geographical status was assessed in the first round in the study, we may not have captured any migration impact of residence change on the frailty status.

## 5. Conclusions

This study presents novel insights into the complexity of the relationship between baseline SDOH, geographical residence, and frailty status, with the identification of specific and potentially modifiable SDOH for frailty prevention. These results lay the groundwork for identifying characteristics of older adults (including social determinants of health) at the highest risks of unmet needs and frailty. With this knowledge, we can improve the sensitivity of screenings to identify potential needs and influence the design of frailty prevention interventions though individual- and system-level support.

## Figures and Tables

**Table 1.** (a) Baseline demographic characteristics of older adults within rural and urban areas in 2011. (b) Baseline health characteristics and frailty status of older adults within rural and urban areas in 2011.

		(a)		
	Total (*n* = 6082)	Urban (*n* = 4949)	Rural (*n* = 1133)	
Variable				*p*-Value
Mean Age (standard error)	75.12 (0.10)	75.16 (0.12)	74.94 (0.23)	0.41
Age				
65–69 years	1154 (28.6)	930 (28.7)	224 (27.7)	0.35
70–74 years	1274 (25.0)	1028 (24.6)	246 (26.8)	
75–79 years	1230 (19.2)	989 (19.0)	241 (19.9)	
80–84 years	1203 (14.5)	991 (14.6)	212 (13.8)	
85+ years	1221 (12.8)	1011 (13.0)	210 (11.8)	
Sex				
Male	2529 (43.6)	2019 (43.1)	510 (46.0)	0.11
Female	3553 (56.4)	2930 (56.9)	623 (54.0)	
Race				
White	4194 (81.2)	3263 (79.3)	931 (89.9)	0.14
Black	1318 (8.1)	1172 (8.7)	146 (5.0)	
Hispanic	352 (6.5)	318 (7.3)	34 (3.0)	
Other	218 (4.2)	196 (4.7)	22 (2.2)	
Education				
Less than High School	1582 (20.7)	1264 (20.2)	318 (23.1)	0.02
High School	1633 (27.0)	1271 (25.9)	362 (32.1)	
Some College	764 (13.9)	630 (13.9)	134 (14.0)	
College Degree or higher	2049 (38.3)	1732 (40.0)	317 (30.8)	
Marital Status				
Married	2914 (54.6)	2314 (53.8)	600 (58.2)	0.01
Separated/Divorced	740 (12.3)	635 (13.0)	105 (9.3)	
Widowed	2058 (27.0)	1685 (26.8)	373 (27.6)	
Never Married	239 (3.7)	210 (4.1)	29 (2.1)	
Living with Partner	125 (2.3)	99 (2.3)	26 (2.7)	
Residence Type				
Community	5753 (94.4)	4667 (94.0)	1086 (96.1)	0.03
Residential Care	329 (5.6)	282 (6.0)	47 (3.9)	
Income				
$0–24,999	1700 (24.7)	1360 (24.1)	340 (27.1)	0.02
$25,000–49,999	818 (14.1)	653 (13.7)	165 (15.6)	
$50,000–74,999	451 (8.7)	377 (8.9)	74 (8.1)	
$75,000–99,999	230 (4.9)	195 (5.1)	35 (4.0)	
$100,000–199,999	235 (5.3)	210 (5.8)	25 (3.0)	
$200,000+	85 (2.0)	79 (2.3)	6 (0.7)	
Missing	2563 (40.4)	2075 (40.1)	488 (41.6)	
Language				
Language other than English	886 (16.7)	787 (18.5)	99 (8.9)	0.002
English	4976 (83.3)	3969 (81.5)	1007 (91.1)	
		(b)		
	Total (*n* = 6082)	Urban (*n* = 4949)	Rural (*n* = 1133)	
Variable				*p*-Value
Insurance				
Medicaid	921 (11.7)	745 (11.6)	176 (12.1)	0.01
Medigap	3100 (54.1)	2456 (52.9)	644 (59.8)	
Tricare	188 (3.3)	164 (3.6)	24 (2.0)	
Medicare only	1873 (30.9)	1584 (32.0)	289 (26.1)	
Healthcare Access				
Has Regular Doctor	5792 (95.4)	4731 (95.8)	1061 (93.8)	0.01
Seen Doctor Last Year	5708 (93.6)	4672 (94.2)	1036 (90.7)	0.003
Support System/Community				
Total hours help per month	47.06 (2.10)	47.63 (2.52)	44.38 (5.21)	0.60
Household size	1.97 (0.02)	1.98 (0.02)	1.90 (0.03)	0.04
Need help with transportation to doctor	2081 (29.8)	1716 (29.9)	365 (29.1)	0.74
Health Conditions				
Current Smoker	450 (15.4)	343 (14.1)	107 (21.1)	<0.001
Cardiovascular Disease	1589 (24.2)	1266 (23.6)	323 (26.9)	0.07
High Blood Pressure	4082 (63.8)	3356 (64.4)	726 (60.7)	0.04
Arthritis	3391 (53.8)	2732 (53.1)	659 (56.8)	0.08
Osteoporosis	1249 (21.1)	1030 (21.6)	219 (19.2)	0.07
Diabetes	1527 (23.6)	1260 (23.9)	267 (22.4)	0.31
Lung Disease	887 (14.7)	719 (14.6)	168 (14.9)	0.87
Stroke	697 (9.8)	545 (9.4)	152 (11.4)	0.07
Dementia	308 (3.8)	266 (4.1)	42 (2.7)	0.04
Cancer	1562 (25.7)	1288 (26.1)	274 (23.9)	0.18
Multimorbidity	4490 (70.7)	3663 (70.6)	827 (70.7)	0.98
Frailty Status				
Robust	1094 (23.0)	881 (23.1)	213 (22.8)	0.73
Pre-Frail	3242 (53.0)	2652 (53.3)	590 (51.9)	
Frail	1746 (24.0)	1416 (23.7)	330 (25.3)	

Continuous and categorical variables were reported as mean (standard error) and frequency (column percentage). Older adults were only included in the demographics data to show the breakdown of residential type. Individuals in residential care were otherwise not included in the analysis. Sample characteristics are described in our previous work https://www.mdpi.com/2673-9259/5/3/27 (accessed on 15 October 2025) [17].

**Table 2.** Association between frailty status and baseline social determinants of health modified by rural/urban status (*n* = 6082).

	Response					
	Pre-Frail Versus Robust (Referent)		Frail Versus Pre-Frail (Referent)		Frail Versus Robust (Referent)	
Variable	OR (SE)	*p*-Value	OR (SE)	*p*-Value	OR (SE)	*p*-Value
Time since baseline interview	0.97 (0.00)	<0.0001	0.99 (0.02)	0.63	0.96 (0.02)	0.06
Age						
Referent: 65–69 years						
70–74 years	1.21 (0.07)	0.0006	0.60 (0.02)	<0.0001	0.73 (0.07)	0.001
75–79 years	1.47 (0.08)	<0.0001	0.86 (0.03)	<0.0001	1.26 (0.10)	0.005
80–84 years	1.12 (0.08)	0.09	0.78 (0.01)	<0.0001	0.87 (0.05)	0.03
85+ years	0.93 (0.28)	0.81	0.90 (0.06)	0.13	0.83 (0.31)	0.63
Sex						
Referent: Male						
Female	0.87 (0.02)	<0.0001	0.99 (0.01)	0.3699	0.86 (0.01)	<0.0001
Race/Ethnicity						
Referent: White						
Black	0.90 (0.24)	0.69	1.32 (0.03)	<0.0001	1.19 (0.29)	0.46
Hispanic	0.11 (0.11)	0.03	0.41 (0.04)	<0.0001	0.04 (0.04)	0.002
Other	1.53 (0.21)	0.002	0.82 (0.13)	0.20	1.26 (0.08)	0.0002
Education						
Referent: Less than High School						
High School	0.92 (0.10)	0.45	1.40 (0.10)	<0.0001	1.30 (0.05)	<0.0001
Some College	0.99 (0.12)	0.92	1.46 (0.07)	<0.0001	1.44 (0.24)	0.02
College Degree or higher	1.37 (0.22)	0.04	1.33 (0.03)	<0.0001	1.82 (0.24)	<0.0001
Income						
Referent: $0–24,999						
$25,000–49,999	1.21 (0.19)	0.21	1.11 (0.02)	<0.0001	1.34 (0.22)	0.07
$50,000–74,999	1.19 (0.20)	0.31	1.35 (0.04)	<0.0001	1.61 (0.24)	0.0014
$75,000–99,999	0.82 (0.36)	0.65	0.94 (0.09)	0.52	0.77 (0.27)	0.45
$100,000–199,999	1.04 (0.13)	0.77	0.77 (0.03)	<0.0001	0.79 (0.12)	0.13
$200,000+	0.78 (0.50)	0.70	1.51 (0.03)	<0.0001	1.18 (0.75)	0.80
Missing	0.87 (0.24)	0.63	1.24 (0.23)	0.24	1.08 (0.50)	0.87
Language						
Referent: Language other than English						
English	0.69 (0.05)	<0.0001	0.79 (0.05)	0.0002	0.55 (0.06)	<0.0001
Healthcare Access						
Insurance						
Referent: Medicare only						
Medicaid	1.07 (0.17)	0.69	1.04 (0.14)	0.77	1.11 (0.07)	0.12
Medigap	0.89 (0.05)	0.03	0.91 (0.04)	0.04	0.81 (0.08)	0.04
Tricare	1.91 (0.35)	0.0004	0.67 (0.07)	<0.0001	1.27 (0.36)	0.40
Has Regular Doctor	0.65 (0.03)	<0.0001	0.91 (0.19)	0.65	0.59 (0.11)	0.003
Seen Doctor Last Year	0.95 (0.10)	0.61	0.84 (0.10)	0.13	0.80 (0.18)	0.30
Support System/Community						
Marital Status						
Referent: Married						
Separated/Divorced	1.12 (0.05)	0.007	0.92 (0.04)	0.06	1.03 (0.03)	0.28
Widowed	1.04 (0.05)	0.40	0.91 (0.16)	0.61	0.95 (0.13)	0.70
Never Married	0.27 (0.03)	<0.0001	0.73 (0.14)	0.12	0.20 (0.03)	<0.0001
Living with Partner	0.59 (0.02)	<0.0001	0.77 (0.10)	0.05	0.45 (0.06)	<0.0001
Household size	1.00 (0.11)	0.99	0.95 (0.02)	0.02	0.95 (0.09)	0.60
Need help with transportation to doctor	0.68 (0.04)	<0.0001	0.89 (0.02)	<0.0001	0.61 (0.02)	<0.0001
Health						
Multimorbidity	1.12 (0.10)	0.22	1.12 (0.04)	0.001	1.26 (0.15)	0.06

Odds ratios and standard errors were reported to establish the impact of the chosen covariates on older adult frailty status. Rural residence was considered the referent.

## Data Availability

Data and analyses are available upon request of the author team.

## Appendix A

**Table A1.** Association between baseline social determinants of health and frailty status without rural interaction term (*n* = 6082).

	Response					
	Pre-Frail Versus Robust (Referent)		Frail Versus Pre-Frail (Referent)		Frail Versus Robust (Referent)	
Variable	OR (SE)	*p*-Value	OR (SE)	*p*-Value	OR (SE)	*p*-Value
Time since baseline	1.12 (0.00)	<0.0001	1.10 (0.01)	<0.0001	1.23 (0.02)	<0.0001
Age						
Referent: 65–69 years						
70–74 years	1.23 (0.07)	0.001	1.87 (0.03)	<0.0001	2.30 (0.11)	<0.0001
75–79 years	1.97 (0.05)	<0.0001	1.82 (0.10)	<0.0001	3.59 (0.14)	<0.0001
80–84 years	3.62 (0.47)	<0.0001	2.51 (0.15)	<0.0001	9.10 (1.74)	<0.0001
85+ years	8.11 (2.20)	<0.0001	2.85 (0.04)	<0.0001	23.13 (6.25)	<0.0001
Urban residence	2.11 (0.91)	0.08	1.66 (0.52)	0.10	3.50 (0.48)	<0.0001
Sex						
Referent: Male						
Female	0.87 (0.02)	<0.0001	0.99 (0.01)	0.37	0.86 (0.01)	<0.0001
Race						
Referent: White						
Black	1.38 (0.25)	0.08	0.71 (0.02)	<0.0001	0.98 (0.21)	0.91
Hispanic	16.75 (17.02)	0.01	2.51 (0.31)	<0.0001	41.96 (38.04)	<0.0001
Other	1.42 (0.37)	0.18	0.97 (0.13)	0.82	1.38 (0.19)	0.02
Education						
Referent: Less than High School						
High School	1.06 (0.06)	0.24	0.71 (0.02)	<0.0001	0.76 (0.02)	<0.0001
Some College	0.82 (0.10)	0.10	0.65 (0.01)	<0.0001	0.53 (0.06)	<0.0001
College Degree or higher	0.53 (0.08)	<0.0001	0.56 (0.04)	<0.0001	0.30 (0.03)	<0.0001
Marital Status						
Referent: Married						
Separated/Divorced	1.16 (0.04)	<0.0001	1.29 (0.05)	<0.0001	1.50 (0.10)	<0.0001
Widowed	1.12 (0.06)	0.02	1.20 (0.08)	0.01	1.35 (0.05)	<0.0001
Never Married	8.65 (1.45)	<0.0001	1.57 (0.09)	<0.0001	13.54 (2.32)	<0.0001
Living with Partner	2.01 (0.09)	<0.0001	1.55 (0.11)	<0.0001	3.11 (0.36)	<0.0001
Income						
Referent: $0–24,999						
$25,000–49,999	0.68 (0.13)	0.04	0.71 (0.05)	<0.0001	0.49 (0.12)	0.004
$50,000–74,999	0.59 (0.11)	0.01	0.55 (0.05)	<0.0001	0.32 (0.09)	<0.0001
$75,000–99,999	0.68 (0.24)	0.28	0.70 (0.12)	0.03	0.48 (0.09)	0.0002
$100,000–199,999	0.43 (0.06)	<0.0001	0.80 (0.13)	0.16	0.35 (0.10)	0.0002
$200,000+	0.53 (0.39)	0.39	0.23 (0.05)	<0.0001	0.12 (0.12)	0.03
Missing	0.72 (0.17)	0.15	0.70 (0.13)	0.06	0.50 (0.21)	0.10
Language						
Referent: Language other than English						
English	1.81 (0.09)	<0.0001	1.38 (0.08)	<0.0001	2.49 (0.27)	<0.0001
Insurance						
Referent: Medicare only						
Medicaid	1.17 (0.18)	0.29	1.17 (0.11)	0.10	1.37 (0.09)	<0.0001
Medigap	1.04 (0.05)	0.37	1.05 (0.08)	0.54	1.10 (0.14)	0.47
Tricare	0.60 (0.11)	0.004	1.75 (0.13)	<0.0001	1.05 (0.25)	0.83
Healthcare Access						
Has Regular Doctor	1.01 (0.08)	0.92	0.92 (0.19)	0.68	0.93 (0.11)	0.54
Seen Doctor Last Year	1.33 (0.06)	<0.0001	1.44 (0.26)	0.04	1.92 (0.43)	0.003
Support System/Community						
Marital Status						
Referent: Married						
Separated/Divorced	1.16 (0.04)	<0.0001	1.29 (0.05)	<0.0001	1.50 (0.10)	<0.0001
Widowed	1.12 (0.06)	0.02	1.20 (0.08)	0.01	1.35 (0.05)	<0.0001
Never Married	8.65 (1.45)	<0.0001	1.57 (0.09)	<0.0001	13.54 (2.32)	<0.0001
Living with Partner	2.01 (0.09)	<0.0001	1.55 (0.11)	<0.0001	3.11 (0.36)	<0.0001
Household size	1.09 (0.10)	0.34	1.09 (0.03)	0.001	1.18 (0.13)	0.13
Need help with transportation to doctor	2.60 (0.16)	<0.0001	2.17 (0.06)	<0.0001	5.64 (0.38)	<0.0001
Health						
Multimorbidity	1.50 (0.10)	<0.0001	1.61 (0.10)	<0.0001	2.41 (0.32)	<0.0001

Odds ratios and standard errors were reported for covariates impacting older adult frailty status, with the rural residence as the referent.

## Abbreviation

The following abbreviation is used in this manuscript:

SDOH: Social determinants of health
